# *N*-[(2-Chloro­phen­yl)sulfon­yl]-2-meth­oxy­benzamide

**DOI:** 10.1107/S1600536813029012

**Published:** 2013-10-31

**Authors:** S. Sreenivasa, B. S. Palakshamurthy, E Suresha, J. Tonannavar, Yenagi Jayashree, P. A. Suchetan

**Affiliations:** a Department of Studies and Research in Chemistry, Tumkur University, Tumkur, Karnataka 572 103, India; b Department of Studies and Research in Physics, U.C.S., Tumkur University, Tumkur, Karnataka 572 103, India; c University College of Science, Tumkur University, Tumkur 572 103, Karnataka, India; d Department of Physics, Karnatak University, Dharwad, Karnataka 580 003, India; e Department of Studies and Research in Chemistry, U.C.S., Tumkur University, Tumkur, Karnataka 572 103, India

## Abstract

The title compound, C_14_H_12_ClNO_4_S, crystallizes with two mol­ecules in the asymmetric unit. The dihedral angles between the benzene rings are 89.68 (1) (mol­ecule 1) and 82.9 (1)° (mol­ecule 2). In each mol­ecule, intra­molecular N—H⋯O hydrogen bonds between the amide H atom and the meth­oxy O atom generate *S*(6) loops. In the crystal, mol­ecule 2 is linked into inversion dimers through pairs of C—H⋯O inter­actions, forming an *R*_2_^2^(8) ring motif. Mol­ecules 1 and 2 are further linked along the *b-*axis direction through C—H⋯π inter­actions. The crystal structure is further stabilized by several π–π stacking inter­actions [centroid–centroid separations = 3.7793 (1), 3.6697 (1) and 3.6958 (1) Å], thus generating a three-dimensional architecture.

## Related literature

For similar structures, see: Gowda *et al.* (2010 ▶); Suchetan *et al.* (2010*a* ▶,*b* ▶, 2013 ▶). For hydrogen-bond motifs see: Bernstein *et al.* (1995 ▶).
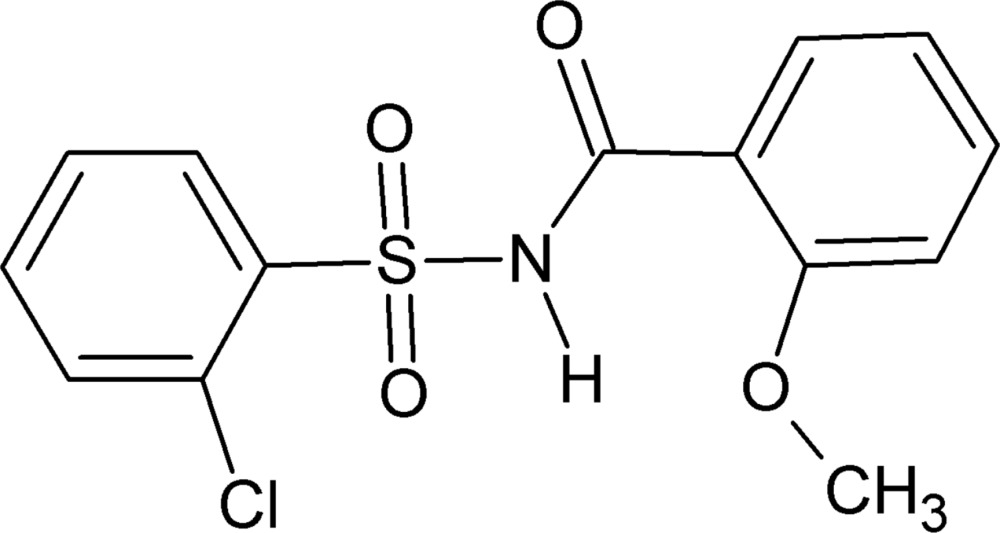


## Experimental

### 

#### Crystal data

C_14_H_12_ClNO_4_S
*M* *_r_* = 325.76
Triclinic, 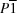

*a* = 8.0508 (3) Å
*b* = 12.9487 (4) Å
*c* = 14.1915 (5) Å
α = 83.897 (2)°
β = 89.368 (2)°
γ = 89.704 (2)°
*V* = 1470.94 (9) Å^3^
*Z* = 4
Mo *K*α radiation
μ = 0.42 mm^−1^
*T* = 293 K
0.36 × 0.29 × 0.23 mm

#### Data collection

Bruker APEXII CCD diffractometer
34023 measured reflections
9760 independent reflections
6518 reflections with *I* > 2σ(*I*)
*R*_int_ = 0.028

#### Refinement

*R*[*F* ^2^ > 2σ(*F* ^2^)] = 0.052
*wR*(*F* ^2^) = 0.163
*S* = 0.90
9760 reflections
389 parameters
H atoms treated by a mixture of independent and constrained refinement
Δρ_max_ = 0.68 e Å^−3^
Δρ_min_ = −0.59 e Å^−3^

### 

Data collection: *APEX2* (Bruker, 2009 ▶); cell refinement: *APEX2* and *SAINT-Plus* (Bruker, 2009 ▶); data reduction: *SAINT-Plus* and *XPREP* (Bruker, 2009 ▶); program(s) used to solve structure: *SHELXS97* (Sheldrick, 2008 ▶); program(s) used to refine structure: *SHELXL97* (Sheldrick, 2008 ▶); molecular graphics: *Mercury* (Macrae *et al.*, 2008 ▶); software used to prepare material for publication: *SHELXL97*.

## Supplementary Material

Crystal structure: contains datablock(s) I, New_Global_Publ_Block. DOI: 10.1107/S1600536813029012/sj5359sup1.cif

Structure factors: contains datablock(s) I. DOI: 10.1107/S1600536813029012/sj5359Isup2.hkl

Supplementary material file. DOI: 10.1107/S1600536813029012/sj5359Isup3.cml

Additional supplementary materials:  crystallographic information; 3D view; checkCIF report

## Figures and Tables

**Table 1:** Hydrogen-bond geometry (Å, °) *Cg* is the centroid of the C22–C27 ring.

*D*—H⋯*A*	*D*—H	H⋯*A*	*D*⋯*A*	*D*—H⋯*A*
N1—H*N*1⋯O4	0.84 (2)	1.97 (2)	2.625 (2)	135 (2)
N2—H*N*2⋯O8	0.83 (2)	1.99 (2)	2.629 (3)	133 (2)
C13—H13⋯O3^i^	0.93	2.50	3.292 (3)	143
C10—H10⋯*Cg*	0.93	2.85	3.729 (3)	157

Symmetry code: (i) 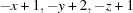.

**Fig. 1.**
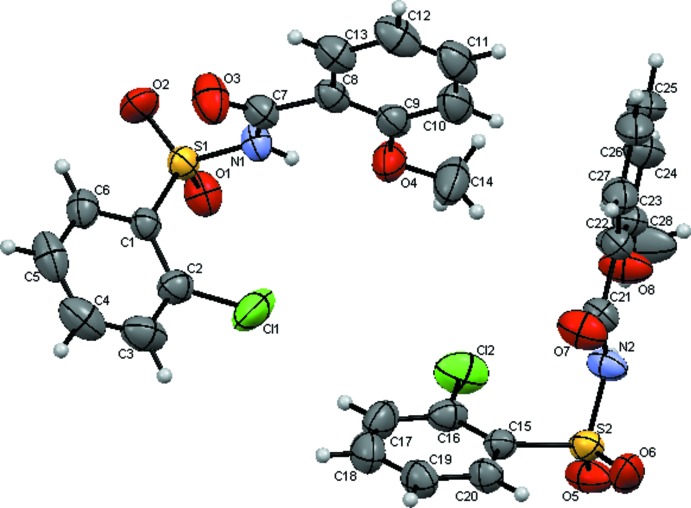
Molecular structure of the title compound, showing the atom-labeling scheme. Displacement ellipsoids are drawn at the 50% probability level.

**Fig. 2.**
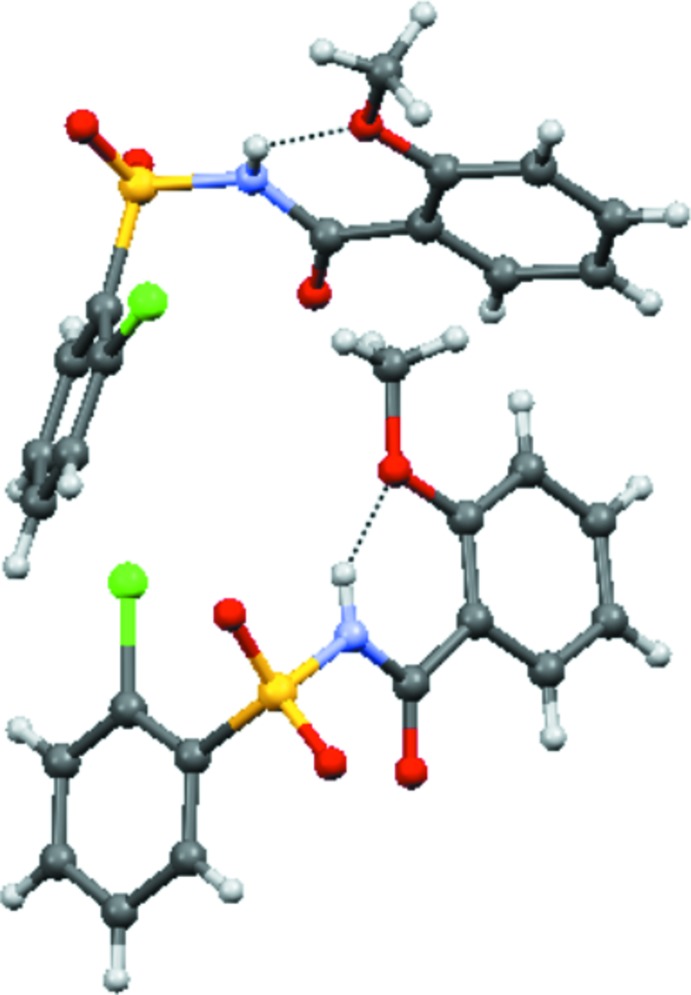
Formation of intramolecular N—H···O hydrogen bonds, dashed lines, generating S(6) loops.

**Fig. 3.**
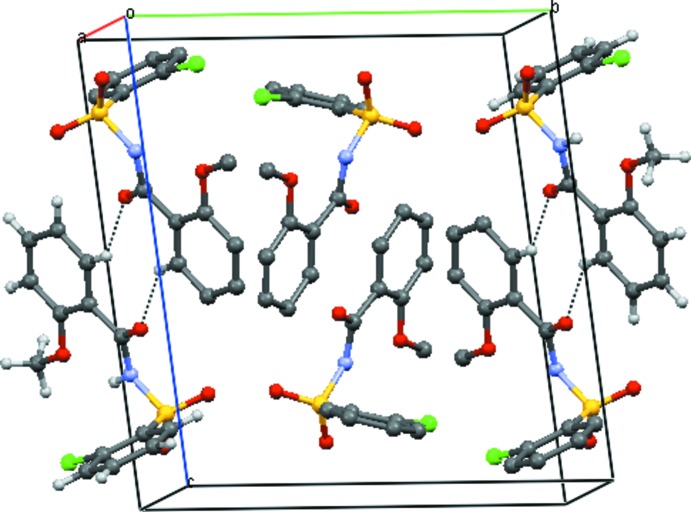
Packing of molecules in I through intermolecular C—H···O interactions, dashed lines, generating R22(8) loops. H atoms not involved in H-bonding are ommitted for clarity.

**Fig. 4.**
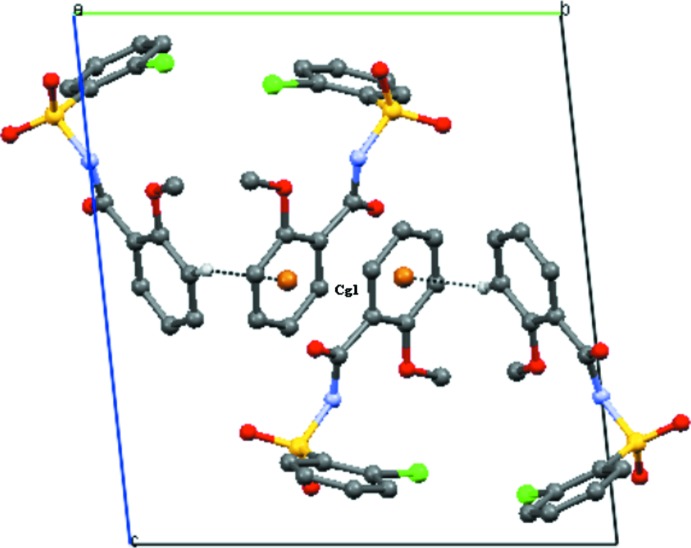
Linking of molecules along b axis in (I) through C—H···Cg1 interactions. H atoms not involved in H-bonding are ommitted for clarity.

**Fig. 5.**
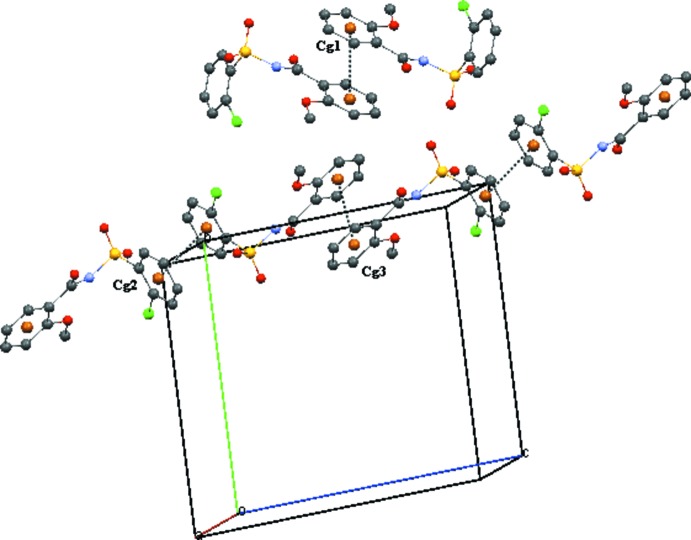
Stacking of molecules in I, through Cg···Cg interactions. Where Cg1 and Cg3 are the centroids of the C8—C13 and C22—C27 methoxy benzene rings and Cg2 is the centroid of the C1—C6 sulfonamide ring. H atoms are ommitted for clarity.

## supplementary crystallographic information

### 1. Comment

As a part of our continued efforts to study the crystal structures of *N*-(aroyl)-arylsulfonamides (Suchetan *et al.*, 2010*a*,*b*,2013), we report here the crystal structure of the title compound (I) (Fig 1).

The title compound (I) crystallizes with two molecules in the asymmetric unit. This is in contrast to the single molecules observed in the asymmetric units of *N*-(benzoyl)-2-chloro-benzenesulfonamide (II) (Gowda *et al.*, 2010), *N*-(2-chlorobenzoyl)-2-chloro-benzenesulfonamide (III) (Suchetan *et al.*, 2010*a*), *N*-(2-methylbenzoyl)-2-chloro-benzenesulfonamide (IV) (Suchetan *et al.*, 2010*b*) and *N*-(3-methoxybenzoyl)-2-chloro-benzenesulfonamide (V) (Suchetan *et al.*, 2013). In the compound, the conformation of the N—H bond in the C—SO_2_—NH—C(O) segment is anti to the C=O bond. The dihedral angles between the two benzene rings in (I) are 89.68 (1)° (molecule 1) and 82.9 (1)° (molecule 2). Compared to this, the dihedral angles are 73.3 (1)° in II (Gowda *et al.*, 2010), 76.9 (1)° in III (Suchetan *et al.*, 2010*a*), 78.7 (1)° in IV (Suchetan *et al.*, 2010*b*) and 87.4 (1)° in V (Suchetan *et al.*, 2013). The conformation of the N—H bond is *syn* to both the *o*-chloro and *o*-methoxy substituents in (I), similar to that observed in V (Suchetan *et al.*, 2013). However, in III (Suchetan *et al.*, 2010*a*) and IV (Suchetan *et al.*, 2010*b*) the opposite effect is observed *i.e.*, the N—H bond is anti to both the *o*-chloro and *o*-methoxy substituents. In both molecules, intramolecular N1—HN1···O4 and N2—HN2···O8 hydrogen bonds between the amide H atoms and the methoxy O atoms, generate S(6) loops (Bernstein *et al.*, 1995)(Fig 2).

In the crystal, molecule 2 is linked into inversion dimers through intermolecular C13—H13···O3 (Fig 3) interactions forming an *R*_2_^2^(8) ring motif (Bernstein *et al.*, 1995). Molecule 1 and 2 are further linked through C10—H10···π interactions along the *b* axis (Fig 4). The crystal structure is further stabilized by several π -π interactions [centroid-centroid separation being 3.7793 (1) Å (for *Cg*1—*Cg*1), 3.6697 (1) Å (for *Cg*3—*Cg*3) and 3.6958 (1) Å (for *Cg*2—*Cg*2)] (Fig 5). *Cg*1 and *Cg*3 are the centroids of the C8···C13 and C22···C27 methoxy benzene rings and *Cg*2 is the centroid of the C1···C6 sulfonamide ring.

### 2. Experimental

The title compound was prepared by refluxing a mixture of 2-methoxybenzoic acid, 2-chlorobenzene sulfonamide and phosphorous oxychloride for 2 h on a water bath. The resulting mixture was cooled and poured into ice cold water. The Solid obtained was filtered and washed thoroughly with water and then dissolved in sodium bicarbonate solution. The compound was later reprecipitated by acidifying the filtered solution with dilute HCl. The filtered and dried solid was recrystallized to the constant melting point (429 K). Colorless prisms of (I) were obtained from a slow evaporation of an ethanolic solution at room temperature.

### 3. Refinement

The H atoms of the NH groups were located in a difference map and later refined freely. The other H atoms were positioned with idealized geometry using a riding model with C—H = 0.93–0.96 Å. All H atoms were refined with isotropic displacement parameters (set to 1.2–1.5 times of the *U*_eq_ of the parent atom).

### Crystal data

**Table:**

C_14_H_12_ClNO_4_S	*F*(000) = 672
*M**_r_* = 325.76	Prism
Triclinic, *P*1	*D*_x_ = 1.471 Mg m^−^^3^
Hall symbol: -P 1	Melting point: 429 K
*a* = 8.0508 (3) Å	Mo *K*α radiation, λ = 0.71073 Å
*b* = 12.9487 (4) Å	Cell parameters from 1234 reflections
*c* = 14.1915 (5) Å	θ = 1.6–31.8°
α = 83.897 (2)°	µ = 0.42 mm^−^^1^
β = 89.368 (2)°	*T* = 293 K
γ = 89.704 (2)°	Prism, colourless
*V* = 1470.94 (9) Å^3^	0.36 × 0.29 × 0.23 mm
*Z* = 4	

### Data collection

**Table:**

Bruker APEXII CCD diffractometer	6518 reflections with *I* > 2σ(*I*)
Radiation source: fine-focus sealed tube	*R*_int_ = 0.028
Graphite monochromator	θ_max_ = 31.8°, θ_min_ = 1.6°
φ and ω scans	*h* = −11→11
34023 measured reflections	*k* = −17→19
9760 independent reflections	*l* = −20→20

### Refinement

**Table:**

Refinement on *F*^2^	Primary atom site location: structure-invariant direct methods
Least-squares matrix: full	Secondary atom site location: difference Fourier map
*R*[*F*^2^ > 2σ(*F*^2^)] = 0.052	Hydrogen site location: inferred from neighbouring sites
*wR*(*F*^2^) = 0.163	H atoms treated by a mixture of independent and constrained refinement
*S* = 0.90	*w* = 1/[σ^2^(*F*_o_^2^) + (0.0876*P*)^2^ + 0.587*P*] where *P* = (*F*_o_^2^ + 2*F*_c_^2^)/3
9760 reflections	(Δ/σ)_max_ = 0.002
389 parameters	Δρ_max_ = 0.68 e Å^−^^3^
0 restraints	Δρ_min_ = −0.59 e Å^−^^3^

### Special details

**Table:**

Geometry. All s.u.'s (except the s.u. in the dihedral angle between two l.s. planes) are estimated using the full covariance matrix. The cell s.u.'s are taken into account individually in the estimation of s.u.'s in distances, angles and torsion angles; correlations between s.u.'s in cell parameters are only used when they are defined by crystal symmetry. An approximate (isotropic) treatment of cell s.u.'s is used for estimating s.u.'s involving l.s. planes.
Refinement. Refinement of *F*^2^ against ALL reflections. The weighted *R*-factor *wR* and goodness of fit *S* are based on *F*^2^, conventional *R*-factors *R* are based on *F*, with *F* set to zero for negative *F*^2^. The threshold expression of *F*^2^ > 2σ(*F*^2^) is used only for calculating *R*-factors(gt) *etc*. and is not relevant to the choice of reflections for refinement. *R*-factors based on *F*^2^ are statistically about twice as large as those based on *F*, and *R*- factors based on ALL data will be even larger.

### Fractional atomic coordinates and isotropic or equivalent isotropic displacement parameters (Å^2^)

**Table:**

	*x*	*y*	*z*	*U*_iso_*/*U*_eq_	
C28	−0.6577 (5)	1.3256 (3)	0.3153 (3)	0.1291 (16)	
H28A	−0.6248	1.2553	0.3352	0.194*	
H28B	−0.6856	1.3319	0.2494	0.194*	
H28C	−0.7526	1.3435	0.3519	0.194*	
HN2	−0.408 (3)	1.5138 (18)	0.2590 (15)	0.057 (6)*	
HN1	0.080 (3)	1.0299 (18)	0.2691 (15)	0.056 (6)*	
C1	0.4099 (2)	1.01693 (13)	0.14113 (11)	0.0413 (3)	
C2	0.3854 (3)	1.11730 (15)	0.09867 (13)	0.0526 (4)	
C3	0.5167 (3)	1.17506 (18)	0.05849 (16)	0.0692 (6)	
H3	0.4993	1.2427	0.0311	0.083*	
C4	0.6705 (4)	1.1329 (2)	0.05921 (16)	0.0732 (7)	
H4	0.7584	1.1720	0.0317	0.088*	
C5	0.7001 (3)	1.0329 (2)	0.09992 (17)	0.0696 (7)	
H5	0.8066	1.0047	0.0995	0.084*	
C6	0.5675 (2)	0.97436 (17)	0.14195 (14)	0.0533 (4)	
H6	0.5857	0.9072	0.1702	0.064*	
C7	0.2633 (3)	1.02032 (16)	0.35657 (13)	0.0504 (4)	
C8	0.1764 (2)	1.07678 (14)	0.42977 (12)	0.0476 (4)	
C9	0.0234 (3)	1.12861 (14)	0.41869 (13)	0.0520 (4)	
C10	−0.0391 (3)	1.18159 (17)	0.49168 (16)	0.0654 (6)	
H10	−0.1393	1.2174	0.4840	0.078*	
C11	0.0456 (3)	1.18137 (18)	0.57482 (17)	0.0701 (6)	
H11	0.0022	1.2170	0.6231	0.084*	
C12	0.1934 (3)	1.1294 (2)	0.58768 (16)	0.0699 (6)	
H12	0.2495	1.1285	0.6447	0.084*	
C13	0.2585 (3)	1.07799 (18)	0.51452 (14)	0.0593 (5)	
H13	0.3598	1.0436	0.5228	0.071*	
C14	−0.2212 (4)	1.1690 (3)	0.3257 (2)	0.0902 (9)	
H14A	−0.2893	1.1400	0.3777	0.135*	
H14B	−0.2682	1.1528	0.2671	0.135*	
H14C	−0.2159	1.2430	0.3260	0.135*	
C15	−0.0676 (2)	1.58600 (13)	0.15496 (11)	0.0407 (3)	
C16	−0.0377 (3)	1.48635 (14)	0.13150 (13)	0.0529 (4)	
C17	0.1220 (3)	1.45853 (18)	0.10457 (17)	0.0687 (6)	
H17	0.1427	1.3924	0.0872	0.082*	
C18	0.2491 (3)	1.5294 (2)	0.10383 (17)	0.0691 (6)	
H18	0.3555	1.5105	0.0859	0.083*	
C19	0.2212 (3)	1.62651 (19)	0.12890 (15)	0.0601 (5)	
H19	0.3083	1.6733	0.1291	0.072*	
C20	0.0624 (2)	1.65527 (15)	0.15405 (13)	0.0485 (4)	
H20	0.0429	1.7219	0.1705	0.058*	
C21	−0.2474 (2)	1.53203 (16)	0.35569 (13)	0.0500 (4)	
C22	−0.3193 (2)	1.45265 (15)	0.42864 (12)	0.0483 (4)	
C23	−0.4520 (3)	1.38669 (16)	0.41651 (13)	0.0527 (4)	
C24	−0.5045 (3)	1.31545 (18)	0.49100 (16)	0.0670 (6)	
H24	−0.5931	1.2716	0.4825	0.080*	
C25	−0.4266 (3)	1.3094 (2)	0.57675 (16)	0.0727 (7)	
H25	−0.4626	1.2614	0.6260	0.087*	
C26	−0.2968 (3)	1.3730 (2)	0.59061 (15)	0.0703 (7)	
H26	−0.2450	1.3690	0.6492	0.084*	
C27	−0.2425 (3)	1.44394 (18)	0.51671 (14)	0.0600 (5)	
H27	−0.1529	1.4866	0.5261	0.072*	
N1	0.1728 (2)	1.00103 (13)	0.27880 (11)	0.0495 (4)	
N2	−0.3337 (2)	1.55288 (14)	0.27293 (11)	0.0526 (4)	
O1	0.11150 (18)	0.93899 (12)	0.12765 (10)	0.0612 (4)	
O2	0.3149 (2)	0.84221 (11)	0.22865 (11)	0.0659 (4)	
O3	0.4060 (2)	0.99214 (16)	0.36392 (12)	0.0797 (5)	
O4	−0.0573 (2)	1.12592 (13)	0.33491 (10)	0.0666 (4)	
O5	−0.37606 (18)	1.61675 (14)	0.10764 (11)	0.0721 (5)	
O6	−0.2480 (2)	1.73358 (11)	0.21074 (12)	0.0707 (4)	
O7	−0.1198 (2)	1.57723 (14)	0.36765 (11)	0.0716 (4)	
O8	−0.5245 (2)	1.39365 (13)	0.32949 (11)	0.0713 (5)	
S1	0.24534 (6)	0.93882 (3)	0.19164 (3)	0.04506 (12)	
S2	−0.26639 (6)	1.63258 (4)	0.18268 (3)	0.04893 (13)	
Cl1	0.19015 (10)	1.17448 (5)	0.09374 (6)	0.0900 (2)	
Cl2	−0.19141 (10)	1.39327 (5)	0.13476 (5)	0.0854 (2)	

### Atomic displacement parameters (Å^2^)

**Table:**

	*U*^11^	*U*^22^	*U*^33^	*U*^12^	*U*^13^	*U*^23^
C28	0.132 (3)	0.133 (3)	0.112 (2)	−0.091 (3)	−0.049 (2)	0.042 (2)
C1	0.0459 (9)	0.0403 (8)	0.0384 (8)	−0.0003 (7)	0.0031 (7)	−0.0087 (6)
C2	0.0644 (12)	0.0439 (9)	0.0494 (10)	−0.0006 (9)	0.0024 (9)	−0.0045 (7)
C3	0.0948 (18)	0.0555 (12)	0.0565 (12)	−0.0177 (12)	0.0091 (12)	−0.0021 (9)
C4	0.0816 (17)	0.0851 (17)	0.0549 (12)	−0.0299 (14)	0.0139 (11)	−0.0172 (11)
C5	0.0465 (11)	0.105 (2)	0.0630 (13)	−0.0006 (12)	0.0042 (10)	−0.0339 (13)
C6	0.0512 (11)	0.0604 (12)	0.0502 (10)	0.0086 (9)	−0.0004 (8)	−0.0147 (8)
C7	0.0534 (11)	0.0536 (10)	0.0443 (9)	−0.0040 (8)	0.0056 (8)	−0.0057 (7)
C8	0.0552 (11)	0.0433 (9)	0.0447 (9)	−0.0114 (8)	0.0098 (8)	−0.0071 (7)
C9	0.0661 (12)	0.0423 (9)	0.0476 (9)	−0.0038 (8)	0.0121 (9)	−0.0061 (7)
C10	0.0828 (16)	0.0524 (12)	0.0623 (12)	0.0013 (11)	0.0169 (11)	−0.0141 (9)
C11	0.0926 (18)	0.0607 (13)	0.0605 (12)	−0.0164 (13)	0.0222 (12)	−0.0239 (10)
C12	0.0870 (17)	0.0763 (15)	0.0491 (11)	−0.0278 (13)	0.0061 (11)	−0.0179 (10)
C13	0.0635 (13)	0.0647 (13)	0.0506 (10)	−0.0142 (10)	0.0051 (9)	−0.0105 (9)
C14	0.0921 (19)	0.103 (2)	0.0768 (16)	0.0482 (17)	−0.0087 (14)	−0.0173 (15)
C15	0.0437 (9)	0.0381 (8)	0.0395 (8)	−0.0020 (7)	0.0014 (6)	−0.0001 (6)
C16	0.0707 (13)	0.0398 (9)	0.0477 (9)	−0.0062 (9)	−0.0028 (9)	−0.0021 (7)
C17	0.0887 (17)	0.0498 (12)	0.0686 (13)	0.0219 (12)	−0.0008 (12)	−0.0119 (10)
C18	0.0566 (13)	0.0793 (16)	0.0706 (14)	0.0147 (12)	0.0058 (11)	−0.0057 (12)
C19	0.0480 (11)	0.0692 (14)	0.0620 (12)	−0.0037 (10)	0.0064 (9)	−0.0027 (10)
C20	0.0475 (10)	0.0454 (9)	0.0525 (10)	−0.0065 (8)	0.0059 (8)	−0.0049 (7)
C21	0.0490 (10)	0.0550 (11)	0.0457 (9)	−0.0053 (8)	0.0039 (8)	−0.0046 (8)
C22	0.0474 (10)	0.0529 (10)	0.0431 (9)	0.0055 (8)	0.0058 (7)	0.0007 (7)
C23	0.0539 (11)	0.0528 (10)	0.0489 (10)	0.0000 (9)	0.0040 (8)	0.0058 (8)
C24	0.0699 (14)	0.0591 (12)	0.0669 (13)	−0.0036 (10)	0.0124 (11)	0.0152 (10)
C25	0.0782 (16)	0.0750 (15)	0.0577 (12)	0.0167 (13)	0.0154 (11)	0.0230 (11)
C26	0.0752 (15)	0.0866 (17)	0.0457 (10)	0.0259 (13)	0.0012 (10)	0.0070 (10)
C27	0.0569 (12)	0.0752 (14)	0.0471 (10)	0.0115 (10)	−0.0006 (9)	−0.0029 (9)
N1	0.0474 (9)	0.0572 (10)	0.0453 (8)	0.0030 (8)	0.0056 (7)	−0.0122 (7)
N2	0.0454 (9)	0.0626 (10)	0.0471 (8)	−0.0139 (8)	0.0024 (7)	0.0072 (7)
O1	0.0529 (8)	0.0724 (10)	0.0619 (8)	−0.0076 (7)	0.0000 (7)	−0.0233 (7)
O2	0.0877 (11)	0.0367 (7)	0.0720 (9)	0.0038 (7)	0.0076 (8)	0.0002 (6)
O3	0.0606 (10)	0.1193 (15)	0.0633 (9)	0.0189 (10)	−0.0076 (8)	−0.0295 (9)
O4	0.0737 (10)	0.0727 (10)	0.0544 (8)	0.0239 (8)	0.0005 (7)	−0.0131 (7)
O5	0.0499 (8)	0.0969 (12)	0.0621 (9)	−0.0079 (8)	−0.0072 (7)	0.0262 (8)
O6	0.0714 (10)	0.0454 (8)	0.0932 (11)	0.0081 (7)	0.0246 (9)	−0.0007 (7)
O7	0.0663 (10)	0.0863 (11)	0.0612 (9)	−0.0286 (9)	−0.0060 (7)	−0.0011 (8)
O8	0.0776 (11)	0.0732 (10)	0.0592 (8)	−0.0338 (8)	−0.0117 (7)	0.0142 (7)
S1	0.0499 (3)	0.0389 (2)	0.0469 (2)	−0.00208 (18)	0.00520 (18)	−0.00783 (16)
S2	0.0410 (2)	0.0494 (3)	0.0531 (2)	−0.00109 (18)	0.00462 (18)	0.00939 (18)
Cl1	0.0898 (5)	0.0554 (3)	0.1200 (6)	0.0259 (3)	−0.0007 (4)	0.0116 (3)
Cl2	0.1187 (6)	0.0528 (3)	0.0861 (4)	−0.0362 (3)	0.0009 (4)	−0.0118 (3)

### Geometric parameters (Å, º)

**Table:**

C28—O8	1.421 (3)	C15—C20	1.381 (2)
C28—H28A	0.9600	C15—C16	1.386 (3)
C28—H28B	0.9600	C15—S2	1.7626 (18)
C28—H28C	0.9600	C16—C17	1.394 (3)
C1—C6	1.381 (3)	C16—Cl2	1.729 (2)
C1—C2	1.387 (3)	C17—C18	1.377 (4)
C1—S1	1.7692 (18)	C17—H17	0.9300
C2—C3	1.381 (3)	C18—C19	1.360 (3)
C2—Cl1	1.733 (2)	C18—H18	0.9300
C3—C4	1.350 (4)	C19—C20	1.383 (3)
C3—H3	0.9300	C19—H19	0.9300
C4—C5	1.381 (4)	C20—H20	0.9300
C4—H4	0.9300	C21—O7	1.208 (2)
C5—C6	1.403 (3)	C21—N2	1.372 (2)
C5—H5	0.9300	C21—C22	1.493 (3)
C6—H6	0.9300	C22—C27	1.394 (3)
C7—O3	1.206 (2)	C22—C23	1.394 (3)
C7—N1	1.375 (3)	C23—O8	1.366 (2)
C7—C8	1.498 (3)	C23—C24	1.391 (3)
C8—C13	1.380 (3)	C24—C25	1.370 (3)
C8—C9	1.402 (3)	C24—H24	0.9300
C9—O4	1.365 (2)	C25—C26	1.363 (4)
C9—C10	1.390 (3)	C25—H25	0.9300
C10—C11	1.369 (3)	C26—C27	1.387 (3)
C10—H10	0.9300	C26—H26	0.9300
C11—C12	1.368 (4)	C27—H27	0.9300
C11—H11	0.9300	N1—S1	1.6464 (16)
C12—C13	1.388 (3)	N1—HN1	0.84 (2)
C12—H12	0.9300	N2—S2	1.6456 (16)
C13—H13	0.9300	N2—HN2	0.83 (2)
C14—O4	1.431 (3)	O1—S1	1.4167 (15)
C14—H14A	0.9600	O2—S1	1.4193 (15)
C14—H14B	0.9600	O5—S2	1.4229 (16)
C14—H14C	0.9600	O6—S2	1.4159 (17)
			
O8—C28—H28A	109.5	C15—C16—Cl2	122.57 (17)
O8—C28—H28B	109.5	C17—C16—Cl2	117.90 (16)
H28A—C28—H28B	109.5	C18—C17—C16	119.7 (2)
O8—C28—H28C	109.5	C18—C17—H17	120.1
H28A—C28—H28C	109.5	C16—C17—H17	120.1
H28B—C28—H28C	109.5	C19—C18—C17	121.0 (2)
C6—C1—C2	119.26 (18)	C19—C18—H18	119.5
C6—C1—S1	118.13 (14)	C17—C18—H18	119.5
C2—C1—S1	122.58 (14)	C18—C19—C20	119.6 (2)
C3—C2—C1	120.8 (2)	C18—C19—H19	120.2
C3—C2—Cl1	117.68 (17)	C20—C19—H19	120.2
C1—C2—Cl1	121.52 (15)	C15—C20—C19	120.67 (19)
C4—C3—C2	119.7 (2)	C15—C20—H20	119.7
C4—C3—H3	120.2	C19—C20—H20	119.7
C2—C3—H3	120.2	O7—C21—N2	120.38 (18)
C3—C4—C5	121.4 (2)	O7—C21—C22	122.59 (18)
C3—C4—H4	119.3	N2—C21—C22	117.03 (17)
C5—C4—H4	119.3	C27—C22—C23	117.87 (18)
C4—C5—C6	119.1 (2)	C27—C22—C21	115.59 (18)
C4—C5—H5	120.4	C23—C22—C21	126.54 (17)
C6—C5—H5	120.4	O8—C23—C24	122.1 (2)
C1—C6—C5	119.7 (2)	O8—C23—C22	117.77 (16)
C1—C6—H6	120.1	C24—C23—C22	120.1 (2)
C5—C6—H6	120.1	C25—C24—C23	120.4 (2)
O3—C7—N1	120.28 (18)	C25—C24—H24	119.8
O3—C7—C8	122.73 (18)	C23—C24—H24	119.8
N1—C7—C8	116.99 (17)	C26—C25—C24	120.7 (2)
C13—C8—C9	118.47 (18)	C26—C25—H25	119.7
C13—C8—C7	115.62 (18)	C24—C25—H25	119.7
C9—C8—C7	125.90 (17)	C25—C26—C27	119.5 (2)
O4—C9—C10	122.6 (2)	C25—C26—H26	120.3
O4—C9—C8	117.88 (16)	C27—C26—H26	120.3
C10—C9—C8	119.5 (2)	C26—C27—C22	121.4 (2)
C11—C10—C9	120.5 (2)	C26—C27—H27	119.3
C11—C10—H10	119.8	C22—C27—H27	119.3
C9—C10—H10	119.8	C7—N1—S1	124.23 (14)
C12—C11—C10	120.8 (2)	C7—N1—HN1	119.7 (15)
C12—C11—H11	119.6	S1—N1—HN1	115.3 (15)
C10—C11—H11	119.6	C21—N2—S2	123.34 (14)
C11—C12—C13	119.1 (2)	C21—N2—HN2	120.8 (15)
C11—C12—H12	120.4	S2—N2—HN2	113.6 (15)
C13—C12—H12	120.4	C9—O4—C14	118.77 (17)
C8—C13—C12	121.5 (2)	C23—O8—C28	118.52 (18)
C8—C13—H13	119.2	O1—S1—O2	118.75 (10)
C12—C13—H13	119.2	O1—S1—N1	104.48 (9)
O4—C14—H14A	109.5	O2—S1—N1	109.88 (9)
O4—C14—H14B	109.5	O1—S1—C1	110.61 (9)
H14A—C14—H14B	109.5	O2—S1—C1	107.38 (9)
O4—C14—H14C	109.5	N1—S1—C1	104.90 (8)
H14A—C14—H14C	109.5	O6—S2—O5	119.47 (11)
H14B—C14—H14C	109.5	O6—S2—N2	109.83 (10)
C20—C15—C16	119.46 (17)	O5—S2—N2	104.50 (9)
C20—C15—S2	116.91 (14)	O6—S2—C15	108.02 (9)
C16—C15—S2	123.60 (14)	O5—S2—C15	108.78 (9)
C15—C16—C17	119.53 (19)	N2—S2—C15	105.37 (8)
			
C6—C1—C2—C3	−0.8 (3)	O7—C21—C22—C23	−170.5 (2)
S1—C1—C2—C3	−178.80 (16)	N2—C21—C22—C23	9.9 (3)
C6—C1—C2—Cl1	179.04 (14)	C27—C22—C23—O8	−178.48 (18)
S1—C1—C2—Cl1	1.0 (2)	C21—C22—C23—O8	0.5 (3)
C1—C2—C3—C4	1.0 (3)	C27—C22—C23—C24	0.3 (3)
Cl1—C2—C3—C4	−178.82 (18)	C21—C22—C23—C24	179.3 (2)
C2—C3—C4—C5	−0.4 (4)	O8—C23—C24—C25	178.7 (2)
C3—C4—C5—C6	−0.5 (3)	C22—C23—C24—C25	0.0 (3)
C2—C1—C6—C5	−0.1 (3)	C23—C24—C25—C26	0.1 (4)
S1—C1—C6—C5	178.03 (15)	C24—C25—C26—C27	−0.5 (4)
C4—C5—C6—C1	0.7 (3)	C25—C26—C27—C22	0.9 (3)
O3—C7—C8—C13	−11.6 (3)	C23—C22—C27—C26	−0.8 (3)
N1—C7—C8—C13	168.32 (17)	C21—C22—C27—C26	−179.84 (19)
O3—C7—C8—C9	167.3 (2)	O3—C7—N1—S1	0.8 (3)
N1—C7—C8—C9	−12.8 (3)	C8—C7—N1—S1	−179.16 (13)
C13—C8—C9—O4	−179.34 (17)	O7—C21—N2—S2	4.3 (3)
C7—C8—C9—O4	1.8 (3)	C22—C21—N2—S2	−176.06 (14)
C13—C8—C9—C10	1.6 (3)	C10—C9—O4—C14	−7.3 (3)
C7—C8—C9—C10	−177.29 (18)	C8—C9—O4—C14	173.6 (2)
O4—C9—C10—C11	179.4 (2)	C24—C23—O8—C28	0.0 (4)
C8—C9—C10—C11	−1.5 (3)	C22—C23—O8—C28	178.8 (3)
C9—C10—C11—C12	0.2 (3)	C7—N1—S1—O1	−179.03 (16)
C10—C11—C12—C13	1.1 (4)	C7—N1—S1—O2	52.54 (18)
C9—C8—C13—C12	−0.3 (3)	C7—N1—S1—C1	−62.61 (17)
C7—C8—C13—C12	178.66 (19)	C6—C1—S1—O1	−128.77 (15)
C11—C12—C13—C8	−1.0 (3)	C2—C1—S1—O1	49.27 (17)
C20—C15—C16—C17	−1.9 (3)	C6—C1—S1—O2	2.24 (17)
S2—C15—C16—C17	175.80 (15)	C2—C1—S1—O2	−179.72 (15)
C20—C15—C16—Cl2	177.69 (14)	C6—C1—S1—N1	119.12 (15)
S2—C15—C16—Cl2	−4.6 (2)	C2—C1—S1—N1	−62.85 (17)
C15—C16—C17—C18	1.6 (3)	C21—N2—S2—O6	−61.0 (2)
Cl2—C16—C17—C18	−178.03 (18)	C21—N2—S2—O5	169.72 (18)
C16—C17—C18—C19	−0.1 (4)	C21—N2—S2—C15	55.13 (19)
C17—C18—C19—C20	−1.1 (3)	C20—C15—S2—O6	−7.14 (17)
C16—C15—C20—C19	0.8 (3)	C16—C15—S2—O6	175.07 (15)
S2—C15—C20—C19	−177.10 (15)	C20—C15—S2—O5	123.94 (15)
C18—C19—C20—C15	0.7 (3)	C16—C15—S2—O5	−53.85 (17)
O7—C21—C22—C27	8.5 (3)	C20—C15—S2—N2	−124.48 (15)
N2—C21—C22—C27	−171.13 (18)	C16—C15—S2—N2	57.73 (17)

### Hydrogen-bond geometry (Å, º)

Cg is the centroid of the C22–C27 ring.

**Table:**

*D*—H···*A*	*D*—H	H···*A*	*D*···*A*	*D*—H···*A*
N1—H*N*1···O4	0.84 (2)	1.97 (2)	2.625 (2)	135 (2)
N2—H*N*2···O8	0.83 (2)	1.99 (2)	2.629 (3)	133 (2)
C13—H13···O3^i^	0.93	2.50	3.292 (3)	143
C10—H10···*Cg*	0.93	2.85	3.729 (3)	157

Symmetry code: (i) −*x*+1, −*y*+2, −*z*+1.
